# Retrospective study of histomorphological examination of skin lesion biopsies and their clinical concordance: experience from a tertiary care hospital in India

**DOI:** 10.25122/jml-2025-0114

**Published:** 2025-11

**Authors:** Tarang Patel, Gyanendra Singh, Vaishali Bhankhodia, Kesha Rachani, Avani Dangar, Jayshukh Berani, Yashdeep Pathania, Krupal Joshi, Deepa Shukla

**Affiliations:** 1 Department of Pathology, All India Institute of Medical Sciences (AIIMS), Rajkot, Gujarat, India; 2 Department of Dermatology, All India Institute of Medical Sciences (AIIMS), Rajkot, Gujarat, India; 3 Department of Preventive and Social Medicine, All India Institute of Medical Sciences (AIIMS), Rajkot, Gujarat, India; 4 Department of Ayurveda, Yoga & Naturopathy, Unani, Siddha, and Homeopathy, All India Institute of Medical Sciences, Jodhpur, Rajasthan, India

**Keywords:** dermatopathology, clinicopathological concordance, inflammatory dermatitis, vesiculobullous diseases, melanocytic lesions

## Abstract

Histopathological examination is a cornerstone in dermatological diagnosis, providing critical insights into the pathogenesis and classification of cutaneous disorders. This study aims to evaluate the concordance between clinical diagnoses and histopathological findings in skin lesion biopsies. This descriptive cross-sectional retrospective study analyzed 321 skin biopsies performed in a dermatology outpatient department. Demographic data, clinical presentations, biopsy site locations, and histopathological findings were systematically recorded. Biopsies were categorized into 12 groups based on histopathological diagnosis, and clinicopathological concordance was assessed. The study included 321 cases (174 men, 147 women) aged 3 to 90 years. Inflammatory dermatitis was the most common diagnosis (39.87%), followed by vesiculobullous diseases (10.90%) and benign keratinocytic/epidermal proliferation (10.59%). The head region was the most frequent biopsy site (20.87%), and plaque formation (30.52%) was the predominant clinical presentation. Overall, clinicopathological concordance was observed in 72.6% of cases. Notably, higher concordance rates were found in melanocytic lesions (100%) and vesiculobullous diseases (85.71%). Chi-square analysis revealed a highly significant association (*P* < 0.001) between the presence of clinical differential diagnoses and concordance with histopathological findings. This study highlights the value of histopathological examination in confirming clinical diagnoses of dermatological conditions, particularly for complex cases. To enhance clinicopathological concordance, dermatologists should provide comprehensive clinical information and, where relevant, include differential diagnoses (D/D).

## INTRODUCTION

Histopathological examination of skin tissue is a fundamental tool in dermatological diagnosis, providing essential insights into the development and classification of skin disorders. This microscopic analysis identifies distinct structural patterns and cellular changes that define various dermatological conditions, facilitating precise diagnosis and targeted treatment strategies. Inflammatory skin disorders account for a significant share of dermatological cases. Psoriasis, for example, exhibits hallmark features such as epidermal thickening with uniform acanthosis, parakeratosis, and neutrophilic collections known as Munro's microabscesses, alongside dilated capillaries in the dermal papillae [1]. In contrast, eczematous dermatitis is characterized by spongiosis, varying degrees of acanthosis, and a predominantly lymphohistiocytic infiltrate [2]. These histological differences are crucial for distinguishing between clinically similar inflammatory conditions.

Infectious causes also reveal characteristic features under microscopic scrutiny. Viral cutaneous infections, like varicella-zoster virus and herpes simplex virus, typically display intranuclear inclusions, ballooning degeneration, and giant cells [3]. Fungal infections show identifiable organisms, highlighted by special stains such as periodic acid-Schiff (PAS) and Gomori methenamine silver (GMS), supporting the use of targeted antimicrobial therapies [4]. Vesiculobullous disorders, a diverse category, involve separation either within or below the epidermis. Pemphigus vulgaris is marked by suprabasal acantholysis and intraepidermal clefts, while bullous pemphigoid presents with subepidermal blisters and infiltrates of eosinophils [5]. Direct immunofluorescence complements these findings by revealing specific immunoreactant deposition patterns.

Cutaneous tumors, encompassing both benign and malignant forms, exhibit unique histological traits. Basal cell carcinoma, the most common skin cancer, appears as nests of basaloid cells with peripheral palisading and a cleft between the tumor and stroma [6]. Squamous cell carcinoma shows irregular clusters of atypical keratinocytes with varying keratinization and invasive behavior [7]. Melanoma is characterized by atypical melanocytes with nuclear irregularity, prominent nucleoli, increased mitosis, and pagetoid intraepidermal spread [8]. Vasculitides affecting the skin span a range of vascular inflammatory patterns. Leukocytoclastic vasculitis, a typical small-vessel vasculitis, features fibrinoid necrosis of vessel walls, neutrophilic infiltration, nuclear fragmentation, and red blood cell extravasation, which aid its placement within the Chapel Hill Consensus framework [9].

Recent developments in dermatopathology have integrated immunohistochemistry, molecular approaches, and digital pathology. Immunohistochemical markers enhance the identification of cutaneous lymphomas, melanocytic lesions, and mesenchymal tumors [10]. Molecular techniques, such as polymerase chain reaction and next-generation sequencing, have uncovered genetic alterations in skin disorders, refining their classification and treatment options [11]. The correlation of clinical and histopathological findings remains vital for diagnostic accuracy. Integrating clinical presentation with morphological patterns and other microscopic features improves diagnostic accuracy, particularly in lichenoid disorders, connective tissue diseases, and granulomatous dermatoses, where overlapping features may be present [12].

The histopathological landscape of skin disorders spans inflammatory, infectious, vesiculobullous, neoplastic, and vascular conditions, each characterized by distinct microscopic features. Continuous advancements in diagnostic technologies, along with an expanding understanding of molecular mechanisms, are enhancing the accuracy of dermatopathological diagnoses and supporting improved patient outcomes through personalized treatment strategies.

## MATERIAL AND METHODS

This was a descriptive cross-sectional study conducted in the dermatology outpatient department (OPD) that focused on histopathological examination of skin diseases via skin biopsies. As this is a retrospective study, the required ethical exemption certificate was obtained from the institute.

Patients who underwent skin biopsies in the dermatology OPD, patients with skin disease confirmed by skin biopsy, and patients with comprehensive clinical and demographic information available were included in the study. Dermatological disorders involving the oral mucosa were also included in the study. Skin disease diagnoses made clinically without performing a skin biopsy, cases without mentioning of biopsy site, cases with incomplete or inadequate biopsy specimens, and patients with insufficient clinical documentation were excluded from the study.

Skin biopsies were performed by trained dermatology clinicians in the outpatient department. Biopsy sites were carefully selected based on clinical suspicion and dermatological assessment. Standard sterile techniques were employed during specimen collection. Biopsy specimens were immediately transferred to the pathology department. Samples were processed in the histopathology laboratory using standardized protocols. Tissue specimens underwent routine processing.

Demographic information was systematically recorded for each patient, including age, gender, biopsy site, and histopathological report. Detailed histopathological findings were meticulously documented, and the diagnosis was confirmed through comprehensive histological analysis. All biopsies were processed by experienced histopathology technicians, and microscopic examination was performed by experienced dermatopathologists. Patient confidentiality was maintained throughout the study. Patient data were anonymized, and all information was securely stored and managed. Statistical analysis was performed using IBM SPSS Version 27 software.

All skin biopsies were categorized according to the coding scheme outlined in Table 1.

**Table 1:** Categories of skin biopsy diagnosis

Category	Code
Inflammatory dermatitis	1
Vesicular bullous diseases	2
Benign Keratinocytic/epidermal proliferation	3
Vasculitis and vascular proliferation	4
Melanocytic lesions	5
Cysts	6
Infectious diseases/granuloma	7
Adnexal tumors	8
Mesenchymal tumors	9
Fibrous/collagen/calcium/histiocytic aggregates	10
Malignant epithelial tumors	11
Descriptive	12

## RESULTS

A total of 321 skin biopsy cases were analyzed, with patients aged 3 to 90 years. Of these, 174 were men and 147 were women, yielding a male-to-female ratio of 1.18:1. The majority of cases were inflammatory dermatitis (128 cases, 39.87%), followed by vesiculobullous diseases (35 cases, 10.90%), benign keratinocytic/epidermal proliferations (34 cases, 10.59%), and infectious or granulomatous dermatitis (23 cases, 7.16%). Other categories included vasculitis and vascular proliferations (19 cases, 5.91%), mesenchymal tumors (12 cases, 3.74%), adnexal tumors (10 cases, 3.11%), malignant epithelial tumors (9 cases, 2.80%), benign cysts (9 cases, 2.80%), non-neoplastic aggregates such as fibrous, collagen, calcium, or histiocytic lesions (8 cases, 2.49%), and melanocytic tumors (4 cases, 1.25%) (Table 2). Most common biopsy site was Head region biopsy 67 cases (including cheek 13, nose 9, eye 2, scalp 9, forehead 5, tongue 5, buccal mucosa 10, lip 7, malar region 1, gingiva 3, ear lobe 2, angle of mandible 1), followed by back 47 cases, forearm 22 cases, leg and arm each having 15 cases and neck 11 cases.

**Table 2:** Distribution of skin lesions by category and gender

Lesion category	Male	Female	No of cases	Percentage %
Inflammatory dermatitis	69	59	128	39.87 %
Vesiculobullous diseases	21	14	35	10.90 %
Benign keratinocytic/epidermal proliferation	13	21	34	10.59 %
Vasculitis and vascular proliferation	9	10	19	5.91 %
Melanocytic tumors	3	1	4	1.25 %
Benign Cysts	4	5	9	2.80 %
Infectious diseases/granuloma	15	8	23	7.16 %
Adnexal tumors	6	4	10	3.11 %
Mesenchymal tumors	7	5	12	3.74 %
Fibrous/collagen/calcium/histiocytic aggregates	4	4	8	2.49 %
Malignant epithelial tumors	5	4	9	2.80 %
Descriptive	18	12	30	9.34 %
Total	174	147	321	100%

The clinical presentations of all categories were classified as patch, plaque, papule, nodule, vesicle/bulla, and macule/purpura. The most common clinical presentation was plaque formation (*n* = 98 (30.52%), followed by papule (*n* = 68, 21.18%), nodule (*n =* 58, 18.06%), vesicle/bulla (*n* = 39, 12.15%), patch (*n* = 38, 11.84%) and purpura formation (*n =* 20, 6.23%; Table 3).

**Table 3:** Clinical presentation in each category

Lesion category	Patch	Plaque	Papule	Nodule	Vesicle/Bulla	Macule/Purpura	Total
Inflammatory dermatitis	11	62	29	15	2	9	128
Vesicular bullous diseases	1	2	1	0	31	0	35
Benign keratinocytic/epidermal proliferation	1	8	10	14	1	0	34
Vasculitis and vascular proliferation	1	7	1	1	0	9	19
Melanocytic lesions	0	2	1	1	0	0	4
Benign Cysts	0	0	3	5	1	0	9
Infectious diseases/ granuloma	11	7	4	1	0	0	23
Adnexal tumors	0	1	5	4	0	0	10
Mesenchymal tumors	3	2	4	3	0	0	12
Fibrous/collagen/calcium/histiocytic aggregates	1	2	1	4	0	0	8
Malignancy	0	0	3	6	0	0	9
Descriptive	9	5	6	4	4	2	30
Total	38 (11.84%)	98 (30.52%)	68 (21.18%)	58 (18.06%)	39 (12.15%)	20 (6.23%)	321 (100%)

Clinicopathological concordance was seen in 218 cases (72.6%). The remaining cases showed discordance, which may be attributed to factors such as non-representative biopsy sites, specimens obtained in early or late disease stages, or biopsies taken after treatment, all of which can alter histopathological appearance (Table 4).

**Table 4:** Concordance of histopathological findings with clinical diagnosis across lesion categories

Category code (Based on microscopy)	*n*	Concordance with clinical data (n)			% of concordant correlation
		With definite diagnosis on HPE	With descriptive diagnosis on HPE	Total	
1	128	81	15	96	75.00%
2	35	24	6	30	85.71 %
3	34	21	4	25	73.53%
4	19	6	5	11	57.89%
5	4	4	0	4	100.00%
6	9	6	1	7	77.78%
7	23	10	8	18	78.26%
8	10	4	2	6	60.00%
9	12	6	2	8	66.67%
10	8	4	4	4	50.00%
11	9	5	1	6	66.67%
Total	291	171	43	214	73.54%

A total of 23 cases were of infectious origin or showed granulomatous dermatitis. Among these, 14 cases were diagnosed as lepromatous leprosy and 4 as tuberculoid leprosy. All leprosy cases were confirmed using Fite–Faraco staining. Additionally, three cases were identified as lupus vulgaris, one as lupus miliaris disseminatus faciei (LMDF), and one as granulomatous dermatitis of unknown etiology.

**Figure 1:**
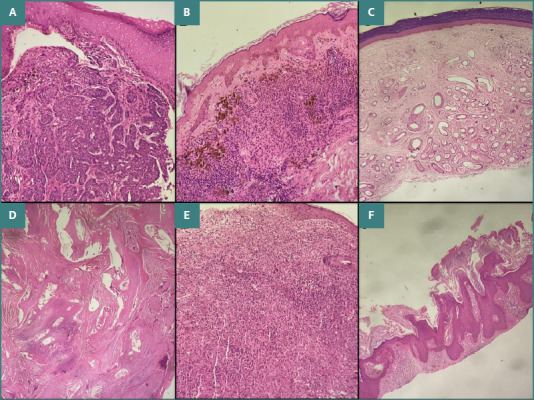
A, Basal cell carcinoma. Section shows stratified squamous epithelium with underlying subepithelium demonstrating a tumor composed of small nests of basaloid cells with peripheral palisading and artefactual clefting (H&E stain, ×40). B, Halo nevus. Section shows infiltration of dermal melanocytes with extensive lymphocytic and histiocytic infiltration (H&E stain, ×20). C, Hemangioma. Section shows epidermis with an underlying lobulated dermal lesion composed of proliferating thin-walled vascular channels (H&E stain, ×40). D, Pilomatricoma. Section shows a well-circumscribed dermal-based lesion exhibiting abrupt keratinization without an intervening granular layer, along with the presence of ghost (shadow) cells and occasional clusters of small basaloid cells (H&E stain, ×40). E, Juvenile xanthogranuloma. Section shows stratified squamous epithelium with dense dermal infiltration of foamy histiocytes and occasional scattered Touton giant cells. Scattered lymphocytes and plasma cells are also present (H&E stain, ×10). F, Seborrheic keratosis, keratotic type. Section shows epidermis with marked orthokeratotic hyperkeratosis and papillomatosis, along with horn pseudocyst formation (H&E stain, ×40).

**Figure 2:**
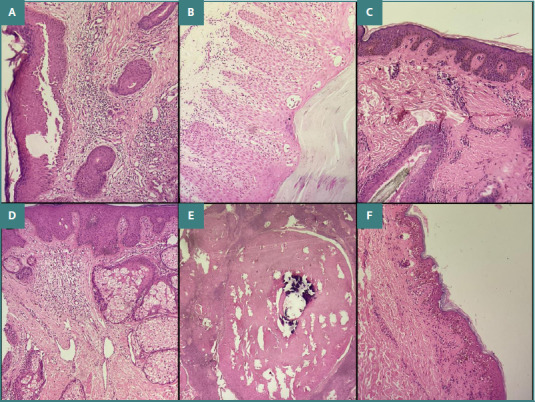
A, Pemphigus vulgaris. Section shows suprabasal acantholysis with an intact basal cell layer (tombstone sign) and intraepidermal vesicles containing round acantholytic keratinocytes (H&E stain, ×40). B, Psoriasiform dermatitis. Section shows hyperkeratosis, regular acanthosis, elongated rete ridges, Munro microabscesses, hypogranulosis, and lymphocytic infiltration within the papillary dermis (H&E stain, ×10). C, Becker nevus. Section shows increased basal layer pigmentation, mild acanthosis, hyperkeratosis, and regular elongation of rete ridges (H&E stain, ×40). D, Nevus sebaceous of Jadassohn. Section shows epidermal acanthosis and elongation of rete ridges with well-formed sebaceous glands extending to and opening on the epidermal surface (H&E stain, ×40). E, Trichilemmal cyst. Section shows a cystic structure with an absent granular layer and thick lamellated keratin within the lumen (H&E stain, ×10). F, Interface dermatitis. Section shows vacuolar alteration of basal cells with sparse perivascular lymphocytic infiltrate (H&E stain, ×10).

**Figure 3:**
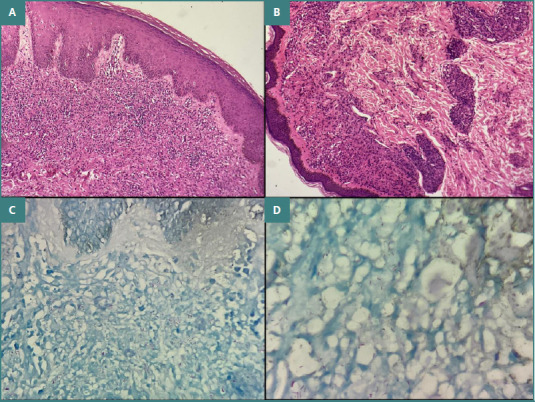
A and B, Lepromatous leprosy. Section shows a prominent grenz zone with macrophages arranged in poorly circumscribed masses within the dermis, containing few lymphocytes. The macrophages are distended with large groups of Mycobacterium leprae bacilli (globi), highlighted in Fite-Faraco stain (H&E stain, ×40). C and D, Fite-Faraco stain demonstrating acid-fast bacilli within foamy macrophages (Fite-Faraco stain, ×40).

The vesiculobullous disease group included cases of pemphigus vulgaris, pemphigus foliaceus, bullous pemphigoid, and dermatitis herpetiformis. Benign epidermal proliferations comprised keratoacanthoma, seborrheic keratosis, verruca vulgaris, nevus sebaceous of Jadassohn, and pseudoepitheliomatous hyperplasia. Adnexal tumors included hidradenoma, sebaceous adenoma, spiradenoma, cylindroma, trichoepithelioma, and trichofolliculoma. Malignant skin tumors consisted of squamous cell carcinoma and basal cell carcinoma (Figures 1–3 A–D).

Clinicopathological concordance with a definitive histopathological (HPE) diagnosis was higher in cases with a clinical differential diagnosis (D/D) than in those with descriptive diagnoses or discordant findings (Table 5). A Chi-square test for independence showed χ^2^ = 52.43 with *P* < 0.001, indicating a highly significant association between the presence of a clinical differential diagnosis and concordance with the HPE diagnosis (Table 6).

**Table 5:** Effect of clinical differential diagnosis(d/d) on clinico-pathological concordance

Clinical d/d	Concordant results with definitive HPE diagnosis *n* = 171	Concordant results with descriptive HPE diagnosis *n* = 43	Discordant results *n* = 107	Total
Present	138	15	48	201
Absent	33	28	59	120
Total	171	43	107	321

**Table 6:** Chi-Square test for independence

Chi-Square Test			
	Value	df	Asymptotic Significance (2-sided)
**Pearson Chi-Square**	52.434^a^	2	<0.001
**Likelihood Ratio**	53.762	2	<0.001
**N of Valid Cases**	321		

a. 0 cells (0.0%) have expected count less than 5. The minimum expected count is 16.07.

## DISCUSSION

Dermatology often relies on skin biopsies to confirm diagnoses, as clinical findings alone can be insufficient for complex cases [13-15]. Histopathology helps diagnose and prognosticate skin diseases, including identifying etiological agents and guiding treatment choices [14,16]. Microscopic examination of skin tissue can reveal subtle changes that are not clinically visible, aiding accurate diagnosis [16].

The correlation between clinical and pathological diagnoses ranges from 67 to 87%. Factors influencing this correlation include the definition of concordance, the pathologist's access to clinical information, and the type of biopsy performed [14]. Providing a comprehensive clinical description and clinical photos can improve diagnostic accuracy [15]. Repeat biopsies can be useful in some cases to achieve an accurate diagnosis [13].

Histopathology helps diagnose granulomatous diseases such as Hansen's disease (leprosy), tubercular granulomas, and non-infectious granulomas. Granulomatous dermatitis is common in the eastern part of India. More than one type of granuloma may show similar histomorphology, and a single pathological lesion may show varied histomorphology. This often leads to confusion regarding accurate diagnosis among pathologists and dermatologists. The most common cause of granulomatous dermatitis is leprosy, including its various subtypes. Other etiologies include tuberculous infection, foreign body granuloma, fungal infection, sarcoidosis, cutaneous leishmaniasis, granuloma annulare, and, more rarely, helminthic infections [17]. Histopathological examination, together with slit-skin smear analysis, plays a crucial role in diagnosing leprosy, while the Ridley–Jopling classification system categorizes its different forms [18,19].

Inflammatory skin diseases are complex and challenging to diagnose, even for experienced dermatopathologists. Histopathology can help confirm the diagnosis of various inflammatory dermatoses characterized by epidermal changes, such as lichenoid dermatitis, spongiotic dermatitis, and psoriasiform dermatitis [20]. Spongiotic dermatitis is characterized by increased intercellular edema and encompasses allergic dermatitis, atopic dermatitis, nummular dermatitis, seborrheic dermatitis, drug reactions, dermatophyte infections, and pityriasis rosea. Spongiotic dermatitis is again subdivided into acute, subacute, and chronic types depending on the timing of biopsy. Lichenoid dermatitis occurs in lichen planus, lichenoid drug reactions, lupus erythematosus, chronic graft-versus-host disease, lichen nitidus, erythema multiforme, pityriasis lichenoides chronica, and mycosis fungoides. Psoriasiform dermatitis is observed in psoriasis, drug eruptions, lichen sclerosus et chronicus, pityriasis rosea, pityriasis rubra pilaris, and pityriasis lichenoides chronica.

Inflammatory dermatoses without epidermal changes are classified according to the predominant inflammatory cell type: lymphocytic, eosinophilic, lymphoplasmacytic, lymphohistiocytic, neutrophilic, or mast cell infiltrates [21].

Vesiculobullous diseases (VBD) of the skin represent a heterogeneous group of dermatoses that have a significant impact on patients and their families, often imposing a substantial economic burden. Immunofluorescence techniques have revolutionized the diagnostic approach to vesiculobullous disorders, serving as a cornerstone for accurate diagnosis and treatment decision-making. Histopathological classification of VBD is based on blister level and inflammation type. The subcorneal group includes pemphigus foliaceus, pemphigus erythematosus, and subcorneal pustular dermatosis, all characterized by very superficial separation just beneath the stratum corneum. The intraspinous category encompasses toxic epidermal necrolysis and erythema multiforme, where cleavage occurs within the spinous layer of the epidermis. Suprabasal blistering disorders, such as pemphigus vulgaris and Hailey-Hailey disease, feature separation immediately above the basal cell layer. Finally, subepidermal blistering conditions—including bullous pemphigoid, dermatitis herpetiformis, and bullous systemic lupus erythematosus—are defined by separation occurring at the dermoepidermal junction. This histopathological classification provides a structured framework for understanding these diverse conditions based on the precise anatomical level of tissue disruption [22].

Benign skin tumors predominate in dermatological practice, typically presenting as well-defined papules, nodules, or cystic formations with smooth borders and a gradual growth pattern. The presence of multiple similar lesions often points towards benignity. In contrast, malignant cutaneous neoplasms tend to appear as isolated lesions with irregular borders and may develop central ulceration as they progress. Benign keratinocytic tumors include various forms of acanthoma, such as keratoacanthoma and seborrheic keratosis, whereas malignant keratinocytic tumors comprise squamous cell carcinoma and basal cell carcinoma. Adnexal tumors are classified based on their differentiation into those of apocrine and eccrine origin, tumors with follicular differentiation, and tumors with sebaceous differentiation [23,24]. Mesenchymal tumors are categorized into fibrous or fibrohistiocytic tumors, vascular tumors, neural tumors, and tumors of muscle origin [25]. Histopathology can differentiate between benign and malignant tumors, with benign lesions showing a clinical-pathological agreement in 68.5% of cases, while malignant tumors show 57.15% concordance [20]. The spectrum of melanocytic tumors can be classified into benign nevi, intermediate melanocytic tumors, and malignant melanoma. Intermediate melanocytic tumors exhibit greater nuclear atypia, mitotic activity, and cellularity than benign nevi [26].

Vasculitis must be differentiated from occlusive vasculopathies based on distinct histopathological features. True vasculitis is characterized by inflammatory cell infiltration within and around the vessel wall, accompanied by evidence of vascular injury. This injury may manifest as leukocytoclasia, endothelial cell necrosis, fibrin deposition, thrombus formation, or degenerative changes in the surrounding connective tissue. Although not universally present, the identification of fibrin is considered a valuable diagnostic indicator of vasculitis.

In contrast, occlusive vasculopathies involve partial or complete vessel obstruction resulting from hypercoagulable states or occurring in association with malignancies, vascular malformations, or hamartomas [27–29]. Vasculitis is further classified into small-vessel and medium-vessel types to refine the diagnosis and guide subsequent diagnostic approaches [30].

The histopathological illustrations presented in our study further confirm the classical diagnostic features observed in lichen planus, lepromatous leprosy, and cutaneous tuberculosis. These findings underscore the indispensable role of histopathology in the accurate diagnosis of dermatological conditions. Our results are consistent with those of other multicenter studies, which have consistently highlighted histopathological examination as a cornerstone for differentiating clinically overlapping dermatoses [31,32].

Potential limitations of this study include its retrospective design, possible selection bias, and variability in biopsy techniques and interpretation. Future research should consider prospective, longitudinal studies with larger sample sizes to provide more comprehensive insights and to better explore correlations between clinical and histopathological findings. It is important to note that a biopsy captures the histopathological features of a lesion at a single point in its evolution; as such, biopsies performed too early or too late in the disease course may yield nondiagnostic results. Clinical diagnosis alone is insufficient; biopsy should be considered essential for complex or atypical cases to ensure diagnostic accuracy.

## CONCLUSION

Histopathological examination proved valuable for diagnosing conditions that pose clinical diagnostic challenges. The highest clinical and pathological concordance is usually present in melanocytic lesions and vesiculobullous lesions. The study underscores the critical importance of comprehensive clinical information and differential diagnoses in facilitating accurate histopathological interpretation of skin lesions. Further prospective studies with larger sample sizes and standardized biopsy techniques are warranted to enhance our understanding of clinicopathological correlations across the spectrum of dermatological disorders.
